# The interplay between EBV and KSHV viral products and NF-κB pathway in oncogenesis

**DOI:** 10.1186/s13027-020-00317-4

**Published:** 2020-10-15

**Authors:** J. Charostad, M. Nakhaie, A. Dehghani, E. Faghihloo

**Affiliations:** 1 Department of Medical Virology, School of Medicine, Ahvaz Jundishapur University of Medical Sciences, Ahvaz, Iran; 2 Department of Microbiology, Shahid Sadoghi University of Medical Science, Yazd, Iran; 3 Department of Medical Microbiology, Kerman University of Medical Sciences, Kerman, Iran; 4 Department of Microbiology, School of Medicine, Shahid Beheshti University of Medical Sciences, Tehran, Iran

**Keywords:** NF-κB, EBV, KSHV, Neoplasm

## Abstract

Among the DNA tumor viruses Epstein-Barr virus (EBV) and Kaposi sarcoma herpesvirus (KSHV), account for a considerable percentage of virus-associated cancers. Deregulation of transcription factors signaling pathways is one of the most significant oncogenic characteristics of EBV and KSHV. NF-κB is a transcription factor that play a remarkable role in oncogenesis because of its function as a master regulator of a spectrum of genes involved in physiological and pathophysiological process. Constitutive activation of NF-κB is a frequent and well-described event in many human malignancies. Compelling evidence represent EBV and KSHV are capable of targeting different components of NF-κB cascade. Here, we summarized recent findings to clarify the precise relationship between dysregulation of NF-κB and EBV and KSHV-related malignancies. This essay also emphasizes on contribution of various viral products in developing cancer through alteration of NF-κB signaling pathway.

## Introduction

Transcription factors (TFs) are the large category of DNA-interacting proteins that bind DNA sequences at specific regulatory elements to either stimulate or inhibit gene transcription via trans-activation or trans-repression domains [1]. TFs are involved in broad range of human disease such as cancers. Nuclear factor-kappa B (NF-κB) is one of the most important member of these groups of proteins which its disruption leads to a wide variety of substantial consequences including inflammation, immune response, cell growth, survival and development of malignant tumors [2]. Novel findings highlighted NF-κB signaling as a paradigm for the source of carcinogenesis that may be deregulated by pathogenic stimuli like viruses [3].

DNA viruses consist of Epstein Barr virus (EBV), Kaposi sarcoma-associated herpesvirus (KSHV), Merkel cell polyomavirus (MCPv) and Human papillomavirus (HPV) which are known to be correlated with several human malignancies [4, 5]. EBV and KSHV are the member of human γ-herpesviruses that are classified as class I carcinogens based on World Health Organization and also account for 2–3 and 10% of all human tumors and infection-related malignancies, respectively [6]. EBV and KSHV are among the tumor viruses that stimulate oncogenesis via hijacking of a series of cell signaling pathways, especially NF-κB [7].

This review attempts to uncover underlying mechanism of EBV and KSHV in developing cancer via NF-κB signaling pathway alteration (Table 1).

**Table 1:** The interactions between viral factors and cellular targets via NF-κB pathway

Virus	Viral Factor	Cellular Target	Mechanism/Effect
	LMP-1	ID1	Induction of ID1 suppresses p16INK4a and promotes cell-cycle progression
		GLUT-1	Elevates glucose uptake and facilitates tumor growth
		hTERT	Activates telomerase and leads to cell immortalization
		DNMT3	Stimulation of DNMT3 downregulates PTEN resulting in Proliferation, invasion, transformation and metastasis
		miR-146a	Modulates interferon response
		HIF-1α	Mediates for survival and progression of tumor cells
EBV		Bmi-1	Derives proliferation and cell survival
		PD-L1	Leads to malignant cell escape
	BRAF-1	Cyclin D1	Elevation of Cyclin D1 represses p21^WAF1^ mediating proliferation and progression of malignant cells
		miR-146a	Activation of miR-146a inhibits SMAD4 inducing cell proliferation and tumor progression
	vFLIP	IMI	Regulates Innate immunity and facilitates KSHV latency
		EZH2	Positive regulation of EZH2 increases ephrin-B2 and induce Angiogenesis
		miR-146a	Enhancement of miR-146a decrease CXCR4 mRNA, facilitating progenitor release of virally infected endothelial cells
KSHV	vGPCR	IL-8, Gro1, IκB, COX-2, cIAP2, and Bcl2	Causes anti-apoptotic response, tumor-associated angiogenesis, cell survival, invasion and inflammation
	miR-K12–1	STAT3/IL-6	Results in tumor promotion

### NF-κB signaling pathway and tumorigenesis

For first time in 1986, NF-κB family transcription factors was discovered by Sen and Baltimore [8] as a B cell­specific TFs. NF-κB is one of the most pleiotropic TFs that its multifaceted remarkable role in physiological functions and pathological conditions such as the cancer development is well-described [9]. NF­κB is constitutively activated in the tumor microenvironment and malignant cells. Constitutive activation of NF-κB triggers upregulation of an vast array of immunity, inflammation, and malignancy-associated genes including apoptotic resistance, invasion, migration, and angiogenesis through upregulating NF-κB responsive genes of cyclooxygenase 2 (COX2), TNF, IL-1,6,8 CXC-chemokine ligands (CXCLs), cyclooxygenase 2 (COX2), vascular endothelial growth factor (VEGF), matrix metalloproteinase (MMPs), B-cell lymphoma (BCLs), cFLIP, ect [10]. It should be noted that some of these upregulated genes, in turn, can target NF-κB. For instance, one of the main upregulated NF-κB downstream target genes is IκBα (a member of IκB family), which terminate NF-κB activation, suggesting of negative reciprocal relationship [9].

**Fig. 1:**
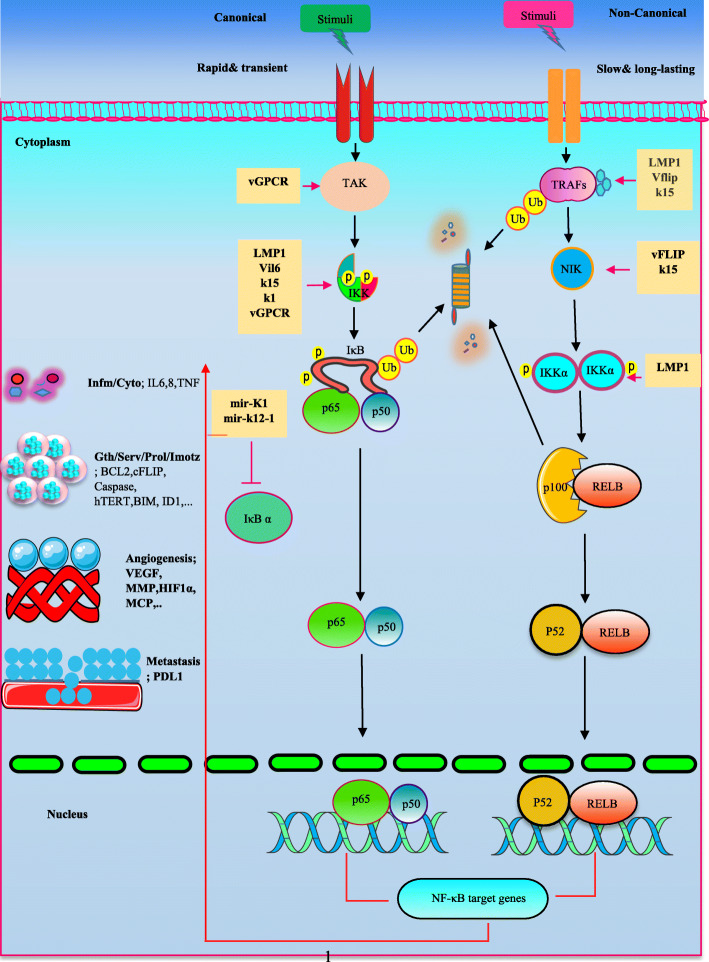
an overview of virus-deregulated NF-κB signaling pathway in oncogenesis. Viral products target different components of NF-κB cascade in order to induce dimers activation (p65,p50 and p52,RELB; other dimers are not shown), driving the transcription of NF-κB target genes following the nuclear translocation. The stimulated genes products can be involved in inflammation, cell growth and cell survival, angiogenesis, metastasis, ect. On the other hand, the NF-κB may lead to production of the factors which negatively regulate the cascade such as IκBα. However, virus might be able to overcome it. For example, KSHV encodes mir-K1 mir-k12–1 to suppress IκBα. Infm/Cyto; inflammation/cytokine, Gth/Serv/Prol/Imotz; growth/survival/proliferation/immortalization

NF-κB family comprises 5 various DNA­binding proteins including NF-κB1(p105-p50), NF-κB2 (p100-p52), Rela (RelA, p65), Rel (c-Rel), and Relb (RelB), forming distinct homodimers and heterodimers to bind promoter of responsive genes on the consensus DNA sequences [11]. The dimers are inactive in an un-stimulated state. RelA, RelB, and c-Rel are retained as a result of their interplay with inhibitor of NF­κB (IκB) proteins in the cytoplasm. In addition, p105 and p100 proteins have conserved C-terminal ankyrin repeats, where enables the proteins to function as IκB proteins. However, C termini of p105 and p100 can undergo of proteasomal degradation and produce p50 and p52 forms, respectively [12]. Activation of NF-κB cascade depends on two unique kinase-dependent pathways, the canonical (classical) and the non-canonical (alternative) NF-κB pathway [13]. The canonical is induced in response to numerous stimuli, including ligands of diverse cytokine receptors, TNF receptor (TNFR) superfamily members, B-cell receptor (BCR),T-cell receptor (TCR) and pattern-recognition receptors (PRRs) [14], that induce NF-κB transiently and rapidly. The receptors receive signals and drive the kinase TGFβ -activated kinase 1 (TAK1; also named MAP 3 K7). TAK1 then activates IκB kinase (IKK) complex, which is composed of three subunits of IKKα (IKK1) and IKKβ (IKK2) as catalytic subunits, and IKKγ (NEMO) as regulatory subunit, by phosphorylation of IKKβ. In next step, IKK complex mediates phosphorylation of IκBα and p105, resulting in their ubiquitination and subsequent proteasomal degradation. NF-κB dimer consists of p50 subunit and RELA /c-REL are bound to IκB proteins, whereas subunits of p50/RELA/c-REL are associated with p105. Eventually, activated dimers translocate to the nucleus, where induce transcription of NF-κB target genes [15–17]. Non-canonical NF-κB activation is slow and long-lasting and occurs upon stimulation of a certain TNFRs superfamily members such as B cell activating factor receptor (BAFFR), CD40, receptor activator of NF-κB (RANK), lymphotoxin-β receptor (LT-βR) [18, 19]. This pathway employs NF-κB-inducing kinase (NIK), an central signaling molecule in the non-canonical pathway. TNF receptor (TNFR)-associated factor (TRAF) proteins are the adaptors that function as a common regulator in canonical and non-canonical NF-κB activation pathways. During the un-stimulated conditions in non-canonical pathway, certain TRAFs-connected NIK is ubiquitinated and proteasomally degraded but upon the receptor stimulation, TRAFs is degraded instead of NIK, resulting in NIK stabilization and accumulation into the cytoplasm [20]. NIK leads to IKKα subunits activation that triggers phosphorylation of p100 and its subsequent processes into p52 via proteasome-mediated degradation. Afterwards, p52 dimerizes with RelB and enters to the nucleus [2]. Tumor viruses such as EBV and KSHV are able to target the various components of NF-κB signaling pathway in favor of their oncogenic properties (Fig. 1).

### Epstein-Barr virus

**Fig. 2:**
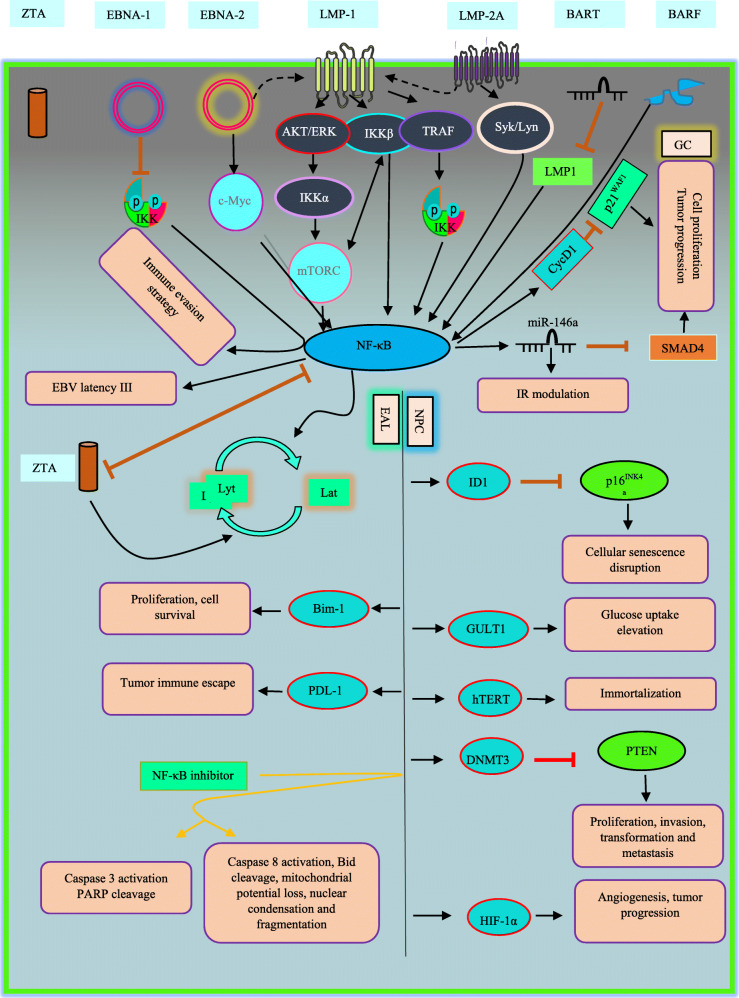
EBV-deregulated NF-κB signaling pathway in oncogenesis. Viral products serve different strategies to activate NF-κB cascade. However, some may induce opposite effect. EAL; EBV-associated lymphomas, Lyt; lytic, Lat; latent

In viruses-associated tumorigenesis, EBV has a remarkable role, the first human virus which was found to be correlated with cancers. EBV is responsible for approximately 1.5 and 1.8% of all cancers and cancer-related deaths, respectively, in the world. EBV is etiologically linked to a broad range of lymphoproliferative disease, lymphoid and epithelial malignancies [21–23]. Generally, EBV establishes two distinct forms of life cycle in the infected cells, namely latent and lytic phase, and EBV oncogenesis is principally associated with latency, which only a limited subset of the full repertoire of viral genes is transcribed [24]. However, the contribution of a limited number of lytic genes is mentioned in the oncogenesis. Latent membrane protein1 and 2A (LMP1,2A) are among the main latent proteins by which EBV can act as driving force in tumorigenesis [25]. LMP1 can regulate host cellular processes through manipulation of cellular signaling pathways including NF-κB that may lead to cell proliferation, cell immortalization and tumorigenesis (Fig. 2) [26]. it is well-documented that LMP-1 initiates the activation of NF-κB cascade [27]. Briefly, LMP1-mediated NF-κB activation upregulates cell surface antigens (CD95, CD54 and CD40), proinflammatory cytokines, angiogenesis elements (COX2 and VEGF) and importantly anti-apoptotic gene products (Bcl-2, A20, cIAP and Bfl-1) [28].

Nasopharyngeal carcinoma (NPC) is an epithelial malignancy which is strongly connected to EBV infection [29]. Since almost all tumors of NPC have displayed NF-κB overexpression, supporting the hypothesis of NF-κB disruption in the process of NPC tumorigenesis [30, 31].^.^LMP1 oncoprotein is found in 80–90% of NPC tumors [32]. Generally, EBV shows latent forms of infection in tumor cells. It has been observed that using NF-κB inhibitors cause EBV reactivation and subsequently cell death in EBV+ vs. EBV-NPC cell lines [33]. p16^INK4a^ acts as limiter element in cell-cycle progression. In response to the stimuli such as activation of oncogenes, p16^INK4a^ promotes cellular senescence [34]. A conducted study in 2004 revealed that this tumor suppressor gene (TSG) was regulated negatively by Inhibitor of DNA Binding/Differentiation 1(ID1), a member of transcription factors involved in cellular immortalization and proliferation in LMP1-expressing nasopharyngeal epithelial cell line [28]. LMP1- activated NF-κB was proposed as mechanism for induction of ID1 expression in the cells. EBV-LMP1 also can modulate process involved in cell energy metabolism to facilitate tumor growth. Elevation in glucose uptake is a major prerequisite for growth of cancer cells which surprisingly, LMP1-mediated NF- κB alters this event. For this purpose, the cells utilize specific glucose transporters such as GLUT-1 to transport extracellular glucose into the cytoplasm [35]. GLUT-1 expression is very low under normal conditions, but is highly expressed in tumorigenesis and considered as a tumor progression marker. Recent study described two underling mechanism for NF-κB-upregulated GLUT-1 in NPC. First, LMP1 affects IKKβ to activate NF-κB and second utilizes mTORC1, an evolutionarily conserved pathway involved in aerobic glycolysis. mTORC1 may either interact with IKKβ and in turn, NF-κB activation or directly interact with it. In addition, mTORC1 activation depends on IKKβ and axis of AKT/ERK/IKK [36]. Independent data also confirmed these results in different type of EBV-related cancer. Et al in 2011 emphasized on dysregulation of GLUT-1 by LMP1-activated NF-κB in B cell lymphomas [37]. Overall, theses pathways alter aerobic glycolysis, a hallmarks of cancers. Driving the cells into immortalization is one of the crucial functions of LMP1 in the context of oncogenesis. LMP1-induced NF-κB is capable of attaching to human telomerase reverse transcriptase (hTERT, involved in apoptotic responses and immortalization [38]) and subsequent translocation into nucleus, Leading to activation of telomerase and cell immortalization in NPC cells [39]. Epstein-Barr nuclear antigen-1 (EBNA1) is a DNA binding protein, where is expressed in all EBV-associated tumors, as a result of its indispensable act in replication and maintenance of the EBV genome [40]. Although EBNA1 have not been considered as a potential oncoprotein by data, its involvement in carcinogenesis in transgenic mice is noted [41]. Dysregulation of biological pathways which are involved in oncogenesis process through EBNA1 is observed. In 2010, Valentine et al. sought whether NF-κB undergoes dysregulation through EBNA1 or not in NPC [42]. They suggested that transient and persistent expression of EBNA1 inhibits IKK phosphorylation and results in repression of NF-κB. Whilst they could not elucidate underlying mechanism of this inhibition. Here, authors concluded this event is a components of immune evasion strategy which is employed by virus.

MicroRNAs (miRNAs) define as small non-coding RNAs which are highly involved in gene expression. Dysregulation of these elements is illustrated in oncogenesis. Depending on certain conditions, miRNAs may act either in favor of oncogenesis or tumor suppression [43]. LMP1 drives miR-146a expression by NF-κB influence on its promoter, subsequently causes negative modulation of interferon response [26]. miR-146a is highly investigated for its multiple-layered role in innate/adaptive immune response and tumorigenesis [44]. EBV is the first human virus that was found to encode miRNAs [45]. BamHI-A rightward transcripts (BARTs) is expressed by EBV. BARTs are multispliced transcripts that its critical role in regulating of cancer-related genes in NPC were documented [46]. BARTs produce various miRNAs in 2 clusters. Fung Lo et al. showed specific types of these miRNA targets the *LMP1* 3 UTR (as a conserved region), leading to negative regulation of LMP-1 protein expression in NPC cells [47]. Additionally, they found BART miRNAs was abundantly expressed in NPC and EBV-infected cell lines and their expression correlates with LMP-1 protein expression inversely in NPC. In this line, they demonstrated Cluster 1 miRNAs introduction into NPC cells suppressed NF-κB activity in dose dependent manner. Importantly, they demonstrated that there was a correlation between amount of LMP-1 and NF-κB activity so that elevation in NF-B activity is followed by increasing amount LMP-1 up to a certain point in HeLa and CNE1 cells. Results suggested that additional LMP-1casused a gradual reduction in activity of NF-κB. surprisingly, they also observed threshold LMP-1 amounts that led to peak of NF-B activity varies in various cells [47]. Authors believe in application of BART miRNAs as a useful strategy for NPC treatment. DNA methylation is one of the main mechanisms of epigenetic modification which may act as a key element in the process of tumorigenesis, when it occurs at particular regions such as promoter of TSGs in human genome [48]. DNA methylation is catalyzed by DNA methyltransferase (DNMT). It is suggested that LMP-1 also can drive epigenetic silencing of tumor suppressor genes via NF-κB. Phosphatase and tensin homolog (PTEN), is a major TSG involved in proliferation, invasion, transformation and metastasis [49]. LMP1 introduction into EBV-negative NPC cells induced NF-κB p65 subunit, where constitutively attached to promoter of DNMT3b and promoted its activation. DNMT3b, in turn, mediated methylation and consequently silencing of PTEN in NPC cells [50]. Low level of O_2_ or hypoxia is a common event in various types of tumors, when the cells proliferate rapidly. Herein, hypoxic conditions induce hypoxia-inducible factor 1 α (HIF-1α) to provide crucial biological processes needed for survival and progression of tumor cells such as angiogenesis [51]. Compelling evidence exhibit that several viral oncoproteins stimulates HIF-1α in human malignancies including EBV-related NPC. Sung et al. in 2016 found that LMP1-mediated NF-κB contributes to upregulation of HIF-1α promoter activity in NPC [52]. They inferred that LMP1serves ERK1/2 signaling pathway for targeting NF-κB as its downstream event to excite HIF-1α gene promoter activity and facilitates tumor progression.

First report of the correlation between EBV and gastric cancers backs to 1990 [53]. EBV is highlighted to be responsible for approximately 10% of all gastric carcinomas (GC) and most prevalent EBV-associated malignancy [54]. BamHI-A rightward frame 1 (BARF1), BARF1 is another EBV-encoded oncogene which selectively expressed in carcinomas and functions as a mitogenic growth factor, inducing immortalization and transformation in epithelial cells [55]. data have confirmed BARF1 is capable of up-regulating NF-κB in gasteric cancers. Elevated level of NF-κB and its target, cyclin D1, was observed in BARF1-expressing gastric cancer cells compared with BARF1 non-expressing cells. Enhanced NF-κB and NF-κB-associated cyclin D1 may diminish p21^WAF1^, as a cell cycle inhibitor, and promotes proliferation and progression of EBV-induced GC [56]. BARF1 mediates positive regulation of miR-146a in NF-κB dependent manner to repress SMAD4, an TSG in stomach cancer, promoting cell proliferation and progression in GC [57].

There is a strong association between EBV infection and lymphoproliferative disorders and malignant lymphomas with origin of B-, T- and NK-cell including Hodgkin, Burkitt and diffuse large B cell lymphomas (HL, BL and DLBCl, respectively) [22]. Proliferative and anti-apoptotic process induced by constitutive activation of NF-κB are considered as two major engaged events in the pathogenesis of HL [58]. Activation of NF-κB Constitutively, is a substantial factor for HL cells survival [59]. Data mentioned this NF-κB activation could be occurred when CD40, a member of TNF receptor family, binds to its ligand (CD154) on HL cells [58]. All BLs express LMP1 and evidence have reflected LMP-1 is able to act as an activated CD40 receptor 55. And utilizes TNFR-associated factors (TRAFs) and the TNFR associated death domain protein (TRADD) to directly stimulates NF-κB [26, 58, 60]. An conducted study introduced that LMP1 up-regulates Bmi-1, an oncogene involved in lymphoid proliferation and cell survival, via NF-κB activation in EB + HL cells, however, the engagement of other NF-κB inducers in order to overexpression of Bmi-1 also have been noted in EBV-HL cells [59]. NF-κB also may target switch from latent to lytic phase in EBV-related malignancies. For instance, EBV LMP1 oncoproteins-elevated NF-κB levels suppress activation of gene promoter, protein synthesis and replication in lytic phase in Burkitt lymphoma [61]. Therefore, switching from the latent to the lytic phase provokes host immune responses against infected cells and may have a remarkable role in killing of tumor cells in the consequence of NF-κB inhibition.

Frequent overexpression of NF-κB is elucidated in several non-Hodgkin’s lymphomas [62]. Diffuse large B-cell (DLBCL), one of the most frequent class of non-Hodgkin’s lymphoma. EBV-associated DLBCLs are defined as an important member of NF-κB-induced aggressive B-cell lymphomas, which high expression level of NF-κB is needed for survival of the lymphoma cells [62, 63]. Gene enrichment and ontology analysis have clarified involvement of NF-κB pathways in EBV(+)DLBCL compared with EBV(−) DLBCL of elderly cases that was in agreement with activation of NF-κB in EBV(+)DLBCL cell lines [64]. EBNA2 takes part in EBV proliferating/growth program (also called latency III) and directly overexpresses LMP1 which, in turn, supporting activation of NF-κB in EBV+ DLBCLs [65, 66]. David et al. demonstrated C-Myc enhances NF-κB-correlated EBV growth program B-cell proliferation when it is subjected to deregulation by LMP1 [63]. C-Myc is a leading TF implicated in cell proliferation and hematological and solid tumors. EBV is detected in a variety of NK and T-cell-related neoplasms including; aggressive NK-cell leukemia (ANKL), NK/T-cell lymphoma nasal type (ENKL), systemic EBV-positive T-cell lymphoma of childhood, and chronic active EBV infection (CAEBV) [67]. EBV + T-lymphoproliferative diseases (EBV + T-LPDs) is categorized into two types: systemic EBV-positive T-cell lymphoma of childhood, an aggressive form, and CAEBV, indolent form. Takada et al. suggested the direct role of EBV infection in persistent activation of NF-κB in T and NK cells related lymphomas. They showed LMP1 expression up-regulate NF-κB activation in a T-cell line, inducing apoptosis protection and immortalization in infected cells [68].

Tumor immune escape is one of the substantial hallmarks of cancers and NF-κB process is tightly related to it. In T cell-mediated immune response, programmed cell death ligand 1 (PD-L1) and its receptor (PD-1) are considered as key checkpoint factors and crucial regulators of tumor immune escape which can be governed by means of NF-κB [69]. On this subject, evidence addressed EBV contribution in tumor immune escape via LMP1- upregulated PD-L1 expression which is mediated by NF-κB activation in Natural killer/T-cell lymphoma (NKTCL), Another EBV-associated lymphoma [70]. Evidence also represent possibility of NF-κB engagement in proliferation, metastasis, invasiveness and chemoresistance and LMP1 may be responsible for aberrant activation of the NF-κB in NKTCL [71–74].

Lymphoblastoid cell lines (LCL), established by EBV *‘*in vitro*’*, are reliable models to understand the precise effect of NF-κB pathway on EBV-transformed cells. Results suggest NF-κB inhibition employs two phase including i) activation of caspase 3 and PARP cleavage and ii) activation of caspase 8, cleavage of Bid, mitochondrial potential loss, nuclear condensation and fragmentation, and to stimulate apoptosis in LCL [75].

As mentioned before, EBV can establish two modes of life cycle in latent and lytic phase. EBV encodes ZTA protein that acts as an activator for lytic cycle cascade [76]. The relationship between ZTA and NF-κB can be defined as a negative reciprocal interaction. Studies report cellular TFs such as NF-κB, Oct-2, Pax-5 and c-Myc interacts with Zta and leads to blockage of EBV lytic reactivation [24]. NF-κB (p65/RelA) is suggested to suppress lytic trans-activator ZTA, promoting the latency. On the other hand, ZTA inhibits NF-kB (p65 subunit) to initiate lytic phase [77]. it seems that biological process determine which one dominates on another in the infected cells.

Although, there are controversial reports on contribution of LMP2A on regulation of NF-κB, considering the fact that LMP2A boosts LMP1 signaling by elevation in LMP1 half-life, representing its indirect positive effect on stimulating NF-κB [78, 79]. Furthermore, existing evidence suggest LMP2A recruits Syk and Lyn kinases to trigger the pathway in direct fashion [60]. on the contrary, in carcinoma cells, LMP-2A presents inhibitory effect on NF-κB [42].

### Kaposi sarcoma-associated herpesvirus

Three types of malignancy comprising primary effusion lymphoma (PEL), multicentric Castleman disease and Kaposi sarcoma (KS) are etiologically linked to Kaposi’s sarcoma-associated herpesvirus (KSHV) [80]. Similar to EBV, during the life cycle of KSHV, the virus exhibits either a latent or a lytic phase that each phase comprises distinct profile of viral gene expression. In Latent phase, virus transcribes a restricted set of viral genes including v-FLIP (k13), LANA, v-Cyclin, K12/Kaposin and viral miRNAs [81]. The virus, also expresses full repertoire of more than 80 genes involved in lytic phase including RTA, K15, K4,K3, K1, vIL- 6, … [82, 83].

Data suggest KSHV latent phase has a direct driving effect on tumor formation and maintenance by hijacking cellular signaling pathways such as NF-κB, while the lytic phase participate in promotion of tumorigenesis in a paracrine manner [84]. In addition, latent and lytic proteins alter NF-κB to promote immune evasion and tumor progression [85].*‘*in vitro*’* and ‘in vivo*’* studies have shown that inhibition of NF-κB can cause reactivaton of KSHV latency form, reducing tumorigenicity in PEL cells and endothelial cell transformation [86]. Sustained activation of NF-κB is observed in all KSHV-infected PEL cells and treatment with NF-κB inhibitors abrogates this activation and induces apoptosis [87].

A number of KSHV products is known to stimulate NF-κB activation. Viral FLICE inhibitory protein (vFLIP) K13 is a key protein in pathogenesis of KSHV-related malignancies [88]. vFLIP, a homolog of cellular FLIP, is a potent inducer of NF-κB pathway that is required for viral latency, survival and tumorigenesis in PEL cell [89]. The interplay between vFLIP and TRAFs, NIK, and IKKs elements provide the stimulating force in order to NF-κB activation [90]. It is worth noting that data provided by Matta and colleagues demonstrated the process of K13-induced NF-κB can be independent from that characterized by inflammatory cytokines (in TRAF6, TAK1 and LUBAC independent manner). In this mechanism, K13 binds to the NEMO subunit, recruiting IKKα and IKKβ and consequent activation via phosphorylation [91]. As a result of NF-κB employment by K13, this protein is able to block lytic replication and alters various cellular processes to modulate cellular survival, proliferation, etc. [88]. In disagreement with these, convincing evidence demonstrated vFLIP may have a reverse impact on NF-κB. vFLIP is capable of elevating A20 expression (as a cellular NF-κB negative regulator). Then A20 binds to NEMO/IKKγ and suppresses NF-κB. In activating state, vFLIP exacerbates cell growth and cytokine production and modulates these process in suppressing state [92]. Of note, this double-edged sword may be in favor of survival and spread of virus in humans, Transient or low activation of NF-κB may results in KSHV lytic replication whereas constitutive or persistent activation of NF-κB may leads to KSHV latency, formation and maintenance of KSHV-induced tumors [86]. In an assessment, introduction of NF-κB inhibitor (Bay 11–7082) initiated oxidative stress which was resulted from up-regulation of reactive oxygen species (ROS), causing virus reactivation and cell death in PEL cells. Importantly, further elevation in ROS and cell death was detected due to usage of NF-κB inhibitor in higher concentration rather than that utilized for inducing virus reactivation [93]. KSHV latency genes also enable the virus to escape from immune system and establish persistency. Furthermore, virus-increased NF-κB acivity is a pivotal factor in repression of KSHV reactivation [93]. KSHV encodes the regulator of transcription activation (RTA) factor to initiate lytic phase in KSHV reactivation [94]. This RTA-induced reactivation may be achieved by decreasing in NF-κB activation. According to positive effect of vFLIP on NF-κB, results show vFLIP is proteasomally degradaded by effect of RTA that leads to NF-κB inactivation [95]. Data exhibited RTA mediates vFLIP degradation via engagement of a particular type of cellular ubiquitin ligase, Itch [96]. In the presence of RTA, Itch is able to potently ubiquitinate vFLIP.

As mentioned earlier, immune response genes can be induced following NF-κB translocation into the nucleus. Interferon regulatory factor 4 (IRF4) is implicated in induction of interferon-stimulated genes (ISG) and innate response. Evidence show vFLIP increases induction of IRF4-mediated ISGs (IMI) through NF-κB activation in PEL cells [97]. IRF4 also found to be a negative stimulator of RTA, thereby facilitating the KSHV latency.

K15 is a KSHV transmembrane protein contributed in proinflammatory and angiogenic pathways, recruiting NIK and IKK α/β in order to activate NF-κB signaling using a specific site in cytoplasmic domain [98]. In lymphocytes, a host regulatory element named paracaspase mucosa-associated lymphoid tissue lymphoma translocation protein-1 (MALT1), which is proposed as a proto-oncogene in lymphomas, regulating antigen receptor-related NF-κB activation by induction of IKK complex activation and cleavage of inhibitory factors of canonical NF-κB (A20 and RelB) [99]. ‘In vitro*’* and xenograft models have been revealed KSHV proteins vFLIP and K15 exploit MALT1 to promote NF-κB activation, driving latency, survival and growth in PEL cell lines [100].

**Fig. 3:**
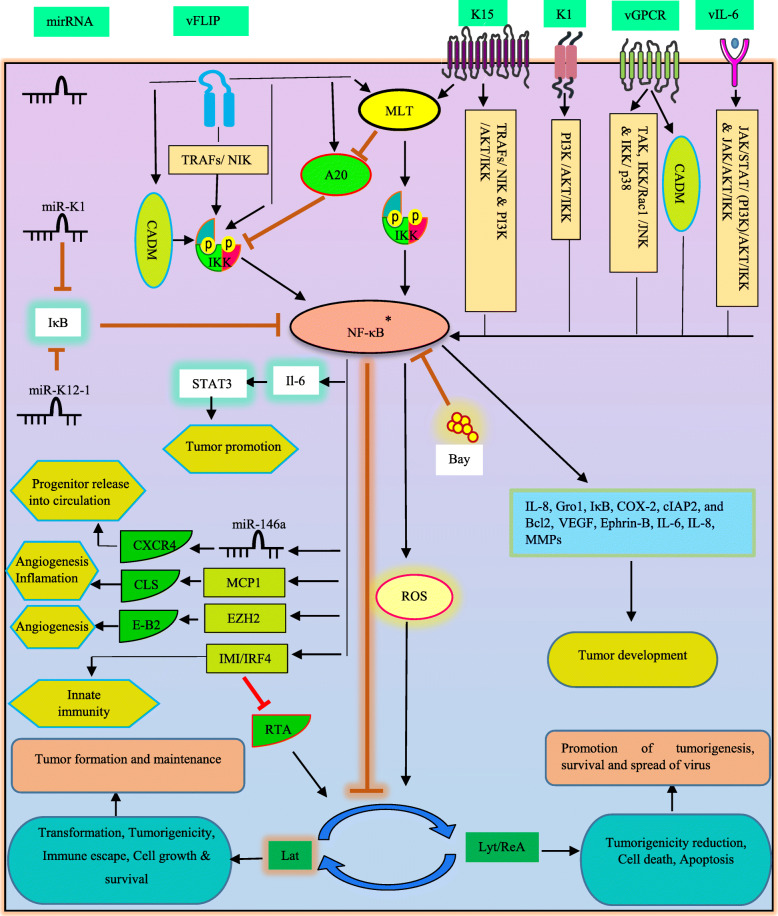
KSHV-deregulated NF-κB signaling pathway in oncogenesis. *Constitutive activation; Transient or low activation of NF-κB results in KSHV lytic replication, whereas constitutive activation of NF-κB may leads to KSHV latency. Virus is capable of engaging signaling molecules in NF-κB activation process. MLT; MALT1, CLS; capillary-like structures, Lat; latent, Lyt/reA; lytic/reactivation

KSHV G protein-coupled receptor (vGPCR) has been emerged as a candidate for KSHV oncogenesis properties and another NF-κB-activating protein [101]. Azzi et al. showed the participation of vGPCR in NF-κB-related cell survival as well as production of proinflammatory cytokines in both PEL cells and KS patients [102]. In addition, they emphasized on influence of vGPCR on communication within microenvironment of tumor and immune response**.** It has been identified that vGPCR causes TAK1, an upstream activator of IKK complex, activation following its phosphorylation and polyubiquitination, accordingly NF-κB induces its target genes such as IL-8, Gro1, IκB, COX-2, cIAP2, and Bcl2 [103]. These elements participate in the process of anti-apoptotic response and tumor-associated angiogenesis, cell survival, invasion and inflammation (Fig. 3). p21-activated kinase 1(Pak 1) is a key oncogenic signaling pathway, frequently involved in cellular processes and human malignancies [104]. KSHV-GPCR may drive Pak1, as an upstream stimulus of NF-κB activity during the cellular transformation in KS tumors [105]. A recent research revealed the crucial effect of cell adhesion molecule 1 (CADM1; involved in cell signaling and tumorigenesis) in survival of PEL cells and KSHV-induced tumorigenesis through chronic activation of NF-κB [101]. vFLIP and vGPCR targeted a special motif at carboxyl terminus of CADM1, maintaining chronic activation of NF-κB. Indeed, CADM1 activated canonical NF-κB through IKK complex.

Angiogenesis is one of the most important hallmarks of KSHV during tumorigenesis. KS is an extremely vascularized and angiogenic tumor of endothelial cell [106]. Relying on evidence, KSHV develops own angiogenic signaling pathways via serving cellular signaling process (in B-cells and endothelial cells), which NF-κB is a remarkable driver in this event. Pathways engaged by KSHV products in order to NF-κB activation may include i) JAK/STAT/ (PI3K) /AKT/IKK and JAK/AKT/IKK for vIL6, ii) PI3K /AKT/IKK for K1&K15, iii) IKK/Rac1 /JNK and IKK/ p38 for vGPCR [107]. Finally, upr-egulation of pro-angiogenic factors (such as VEGF, Ephrin-B, IL-6, IL-8, MMPs and ect) is achieved as a result of NF-κB activation. He et al. found a connection between NF-κB and EZH2 in order to induction of pro-angiogenic factors [108]. The probable role of NF-κB was proposed in elevation of Enhancer of Zeste Homolog 2 (EZH2, a part of polycomb complex) expression by vFLIP and LANA in KS tumors. EZH2, in turn, increased Ephrin-B2. *‘*In vitro*’* model of KSHV infection in endothelial cells revealed virus-triggerd IKK/NF-κB pathway may be noted as a source of formation of capillary-like structures upon the acute infection [109]. MCP-1, as a chemokine detected to be angiogenesis and inflammation mediator, was strongly synthetized in response to the triggered pathway and drove pathogenic angiogenesis and inflammation in KSHV-associated lesions.

KSHV miRNAs are consist of 12 pre-miRNAs which are processed to final 25 mature miRNAs [110]. KSHV miRNAs-NF-κB interplay can govern viral replication. miR-K1 encoded by KSHV ORF-75, directly target 3′ UTR of IκBα transcript and promotes positive regulation of NF-κB and lytic replication inhibition [111]. Similar to EBV, KSHV oncoproteins alters cellular miRNAs in favor of tumorigenesis. vFLIP-induced NF-κB upregulates miR-146a. miR-146a, in turn, transcriptionally suppresses CXCR4 mRNA, facilitating premature release of progenitors of virally infected endothelial into the circulation [88]. Additionally, NF-κB can show its multifaceted role by engaging other signaling proteins such as STAT3 which is implicated in KSHV tumorigenesis [112]. In this line, it has been disclosed that miR-K12–1 serves NF-κB/IL-6/STAT3 signaling in PEL cells. miR-K12–1 act as a IκBα silencer, resulting in NF-κB activation. Besides, elevated level of STAT3 associates with IL-6 upregulation, as a transcriptional target of NF-κB [86].

On the basis of the close relationship between oncogenic viruses and inhibition of TSGs in the development of malignancies, KSHV may involve TSGs to affect NF-κB. PDZ-LIM domain-containing protein 2(PDLIM2) TSG is an essential element for terminating NF-κB activation that performs its role through proteasomal degradation of NF-κB nuclear activated forms [113]. Suppression of PDLIM2 has been found to be correlated to persistent activation of NF-κB in multiple tumors. A conducted study in 2015 revealed transcriptional suppression of PDLIM2 by KSHV leads to NF-κB and STAT3 activation and consequently tumorigenesis and tumor maintenance in KSHV-transformed endothelial cells and cancer cell lines [84]. The study introduced epigenetic modification (DNA methylation) as a cause of PDLIM2 repression. We previously highlighted the importance of DNA methylation in virus-induced TSGs suppression [48].

## Conclusion

Oncogenic γ-herpesviruses EBV and KSHV are the causative agent of multiple human malignancies with origin of lymphoid and epithelial. EBV and KSHV modulate transcription of cancer-related genes via affecting transcription factors. NF-κB is a pivotal transcription factor, governing cancer-related biological pathways such as apoptosis, proliferation, differentiation, angiogenesis and metastasis. EBV and KSHV have evolved special strategies to activate NF-κB constitutively in the course of oncogenesis. The viruses encode proteins by which either directly target different parts of NF-κB cascade or through serving other mediators and signaling pathways, leading to sustained the NF-κB activation. However, some encoded products have been reflected to act against NF-κB activation. Hence, further precise investigation is needed to expand our understanding of the role of NF-κB in γ-herpesviruses-associated cancers.

Accumulating data provide insight into preventing NF-κB activation can be proposed as a promising candidate in cancer therapy strategy. Therefore, developing an anti-cancer treatment regimens based on NF-κB and viral products inhibitors may be a beneficial approach in virus-associated cancers.

## Data Availability

Please contact author for data requests.
